# Tumor Necrosis Factor-Alpha Signaling May Contribute to Chronic West Nile Virus Post-infectious Proinflammatory State

**DOI:** 10.3389/fmed.2020.00164

**Published:** 2020-04-30

**Authors:** A. Arturo Leis, Marie F. Grill, Brent P. Goodman, Syed B. Sadiq, David J. Sinclair, Parminder J. S. Vig, Fengwei Bai

**Affiliations:** ^1^Center for Neuroscience and Neurological Recovery, Methodist Rehabilitation Center, Jackson, MS, United States; ^2^Department of Neurology, Mayo Clinic, Scottsdale, AZ, United States; ^3^Mississippi Baptist Medical Center, Jackson, MS, United States; ^4^Departments of Neurology, Neurobiology, and Biochemistry, University of Mississippi Medical Center, Jackson, MS, United States; ^5^Department of Biological Sciences, University of Southern Mississippi, Hattiesburg, MS, United States

**Keywords:** West Nile virus, fever, immunity, neuroinvasive disease, cytokines, tumor necrosis factor, neuroinflammation

## Abstract

**Background:** West Nile virus (WNV) causes a spectrum of human disease ranging from a febrile illness (WNV fever) to severe neuroinvasive disease (meningitis, encephalitis, acute flaccid paralysis). Since WNV gained entry into North America in 1999, clinicians caring for WNV survivors have observed persistent neurological symptoms occurring long-after the production of neutralizing antibodies and clearance of the virus. Accordingly, alternative pathogeneses other than direct viral invasion have been hypothesized to explain these post-infectious symptoms. The dominant hypothesis is that antiviral inflammatory responses triggered initially to clear WNV may persist to promote a *post-infectious proinflammatory state*.

**Methods:** In 4 serologically-confirmed WNV patients with persistent post-infectious symptoms (3 WNV fever, 1 neuroinvasive disease), we ordered a comprehensive cytokine panel at weeks 8, 10, 12, and 36 months post-onset of illness, respectively, to better understand the pathophysiology of the protracted symptoms.

**Results:** All patients had abnormally elevated tumor necrosis factor alpha (TNF-α), a major molecule triggering antiviral cytokines and chronic inflammation in many human autoimmune diseases, but heretofore not reported to be upregulated in human WNV infection. Three patients also had elevations of other proinflammatory proteins. Major symptoms included fatigue, arthralgias, myalgias, generalized or multifocal pain or weakness, imbalance, headaches, cognitive problems, and symptoms of dysautonomia.

**Conclusion:** The findings provide support for an extended post-infectious proinflammatory state that may contribute to chronic inflammation and long-term morbidity in some WNV survivors and further suggest that TNF-α may play a pathogenic role in initiating this inflammatory environment. Clinical trials may be warranted to determine if TNF-α inhibitors or other immunosuppressive agents can improve patient outcomes.

## Introduction

West Nile virus (WNV) is a neurotropic flavivirus that causes a spectrum of human disease ranging from a febrile illness (WNV fever) to severe neuroinvasive disease classified as meningitis, encephalitis, or acute flaccid paralysis (poliomyelitis variant) (1). In neuroinvasive disease, neuronal loss attributed to direct viral invasion of neurons is the presumed pathological substrate for the high morbidity and mortality. However, since WNV gained entry into North America in the New York City outbreak of 1999 (2), clinicians caring for WNV survivors, including those with WNV fever, have observed persistent symptoms and delayed neurological deficits long after the production of neutralizing antibodies and clearance of the virus. In fact, from 1999 to late 2002, a total of 2,671 cases of human illness were reported to the Centers for Disease Control and Prevention (CDC) and all attempts to recover WNV from these patients failed (3), suggesting that once neutralizing antibodies are formed the virus is rapidly cleared. To date, it is commonly accepted that infectious WNV cannot be isolated from humans with a normal immune system following the production of neutralizing antibodies (4, 5). There is also no convincing evidence that WNV can establish a persistent infection or remain dormant in the human body after the primary infection. Accordingly, alternative pathogeneses other than direct viral invasion have been hypothesized to explain the protracted or delayed post-infectious symptoms, which occur in up to 50% of convalescent patients (6). On a molecular level, acute WNV infection induces a significant upregulation of various proinflammatory proteins, including astroglial protein S100B and the receptor for advanced glycation end products (RAGE) (7), high-mobility group box-1 (HMGB1) (8), and osteopontin (OPN) (9), which initiate and maintain an inflammatory cascade that increases production of pro-inflammatory cytokines, interleukins (ILs), and chemokines (10–12) as part of the initial antiviral immune response. Previous studies in mice and cell cultures have demonstrated essential protective roles for these proinflammatory proteins against acute WNV infection (13, 14). However, these antiviral molecules have also been reported to persist for months following the acute illness, leading to a post-infectious proinflammatory state that may contribute to long-term neuroinflammation and cytotoxicity in some WNV survivors (15, 16).

In 4 patients with persistent post-WNV symptoms, we ordered a comprehensive cytokine panel to better understand the pathophysiology of these protracted symptoms. Three were classified as WNV fever and one as neuroinvasive disease by the Mississippi State Health Department according to clinical features based on criteria established by the Centers for Disease Control and Prevention (CDC) (1). In all, the cytokine panel was collected long after the virus had presumably been cleared by the immune system (at weeks 8, 10, 12, and 36 months post-onset of illness). All 4 patients had elevated levels of TNF-α. Heretofore, upregulation of TNF-α has not been reported in human WNV clinical infection despite general recognition that TNF-α is a major molecule triggering antiviral cytokines in neuroinflammation and many other human autoimmune diseases (10, 13, 17). Three patients also had elevations of other proinflammatory cytokines. These findings provide additional support for an extended post-infectious pro-inflammatory state that may contribute to chronic neuroinflammation and long-term morbidity in some WNV survivors and further suggest that TNF-α may play a pathogenic role in initiating this proinflammatory environment.

## Methods

This retrospective case series was approved by the Institutional Review Board for Human Research at Methodist Rehabilitation Center. Patients also gave approval for their case reports to be published in medical journals. All 4 patients lived in Mississippi and developed an acute febrile illness during the summer with onset of symptoms in August 2017 (2 cases), August 2018 (1 case) and September 2014 (one case). All had a positive WNV IgM antibody in the context of the febrile illness and developed persistent severe post-infectious symptoms. In all, we ordered a commercially available comprehensive cytokine panel (Mayo Clinic test I.D. FCYTP; ARUP Laboratories I.D. 0051394) to better understand the immune physiology of the protracted symptoms. Quantitative multiplex bead assay was used to identify the cytokines. Patients' sera were collected at weeks 8, 10, 12, and 36 months post-onset of illness, respectively. The cytokine panel included TNF-α, interferon gamma (IFN-γ), IL-2, IL-2 receptor (CD 25) soluble, IL-12, IL-4, IL-5, IL-10, IL-13, IL-17, IL-1-beta, IL-6, IL-8, and protein S100B. Cytokines on this panel are key regulators of innate and adaptive immune responses. IL-2, IL-6, IL-12, and IFN-γ have also been reported to be increased in WNV patients with prolonged post-infectious symptoms (16). IL-17 is upregulated by WNV infection in humans and also plays a vital role in the pathogenesis of other human autoimmune diseases (12). In addition, WNV infection significantly upregulates S100B, an important proinflammatory glial protein that is increased in human serum and cerebrospinal fluid (CSF) post-WNV infection (7). S100B binds to the receptor for advanced glycation end products (RAGE), which promotes and amplifies autoimmune and chronic inflammatory diseases (7, 8). WNV activated macrophages also produce a large number of antiviral cytokines found on this panel, including TNF-α, INFs, IL-6, IL-8, and IL-1-beta (18). Importantly, all patients attributed their lingering symptoms to WNV infection.

### Case Series

#### Patient 1

A 49-year-old law enforcement officer was in relatively good health prior to WNV infection with treated depression and hypertension and a history of isolated surgery for a rectal abscess. He developed high fever (104°C), chills (“trembling like a wet noodle”), confusion, weakness in both lower limbs, and urinary retention. He was initially diagnosed as “serotonin syndrome” because he was on fluoxetine, but the medical team revised the diagnosis to recurrent rectal abscess with sepsis (“because he looked so toxic”) and requested surgical intervention, despite a normal white cell count. Under general anesthesia, the surgeon found no evidence of recurrent rectal abscess and informed the patient that “the abscess is not what made you sick, there's something else going on.” Upon tracheal extubation the patient had a respiratory arrest requiring mechanical ventilation for 3 days. Persistent symptoms over the ensuing 8 weeks included disabling fatigue (“still feeling awful”), imbalance, generalized and focal weakness, increased anxiety, restlessness, tremors, generalized pain (“whole body”), and additional pain in joints (“achy painful joints everywhere”). On neurological examination there was mild weakness in both lower limbs (Medical Research Council, MRC, scale 4/5) and left upper limb (4/5) with myoclonic-like jerks, bilateral hand tremors, slight dysmetria on finger-nose testing and slight gait ataxia with several near-falls during tandem gait. He was diagnosed with WNV neuroinvasive disease. A cytokine panel performed 8 weeks after onset of symptoms showed elevated TNF-α with equivocal S100B (Table S1). Normal laboratory tests included complete blood count (CBC), C-reactive protein (CRP), erythrocyte sedimentation rate (ESR), antinuclear antibody (ANA), complete metabolic panel (CMP), and paraneoplastic panel (the latter panel was ordered because of a 36-lb weight loss and a strong family history of cancer). Repeat cytokine panel 21 months after onset of symptoms was normal with TNF-α <5 pg/mL and S100B (76 ng/L).

#### Patient 2

A 53-year-old woman with a history of asymptomatic alpha 1-antitrypsin deficiency and Hashimoto's thyroiditis developed an acute febrile illness with maculopapular rash and neck stiffness diagnosed as WNV fever. This was followed by persistent symptoms for 10 weeks (“still feeling awful”), including extreme fatigue, insomnia, imbalance, dizziness, persistent low grade fever, difficulty concentrating, daily headaches, paresthesias in limbs, muscle and joint pains, and anxiety and depression (“my life as a teacher has turned upside down”). Cytokine panel, performed 10 weeks after onset of symptoms showed elevated TNF-α and interleukin (IL)-2 receptor (CD 25) soluble protein with equivocal S100B (Table S1). The ESR was slightly elevated at 28 mm/h. Neurological examination was unremarkable. The patient was placed on short-term disability for 4 months, but she was “unable to function as a school teacher” because of the extreme fatigue and difficulty concentrating. Repeat cytokine panel 20 months after onset of symptoms showed a normal TNF-α (14 pg/mL) but increased soluble IL-2 receptor (CD 25) to 1571 pg/mL, a cytokine that reflects ongoing immune activation and inflammation (19). Disability was extended for an additional 3 months (total 7 months) before she was able to return to work.

#### Patient 3

A 34-year-old woman in relatively good health except for occasional migraine headaches and chronic nonspecific “colitis” developed an acute febrile illness with maculopapular rash in face and limbs diagnosed as WNV fever. Acute symptoms persisted for 12 weeks, including severe fatigue, weight loss, hand tremors (“I can barely write my name”), difficulty with “hand/eye coordination,” severe muscle aches in calves and thigh muscles, joint pains “all over,” and right lower limb muscle “jerking and jumping.” Inspection of several videos provided by the patient confirmed involuntary repetitive right quadriceps myoclonic-like jerks involving the entire length of the muscle. Dysautonomic symptoms were prominent and included anxiety, a feeling of total body hyperexcitability “with my legs shaking when I walk,” palpitations, irregular heart rate and postural lightheadedness and orthostatic tachycardia attributed to dehydration by her physicians. She also developed severe diarrhea in the month following the acute WNV illness with negative stool cultures that was attributed to a “flare-up” of her colitis. Prior to WNV, the colitis had been well controlled with only mesalamine (5-aminosalicylic acid) for the prior 2 years. She was treated with Flagyl (metronidazole) for 2 weeks for possible infectious colitis and repeat cultures a month later identified Clostridium difficile. A cytokine panel, performed 12 weeks after onset of symptoms, showed elevated TNF-α and IFN-γ (Table S1). Initial neurological examination showed focal weakness (4/5) in right anatomical leg and thigh muscles associated with relative hyporeflexia. Electrodiagnostic studies showed decreased cumulative compound muscle action potential (CMAP) amplitudes from right peroneal, tibial and femoral nerves (26.9 mV) compared with 37.7 mV on the left. In contrast, cumulative sensory nerve action potential (SNAP) amplitudes from sural, superficial peroneal and saphenous nerves were symmetrical (66 μV on the right and 60 μV on the left). Needle EMG showed fibrillation potentials in multiple muscles of the right lower limb including tibialis anterior, medial and lateral gastrocnemius, quadriceps, and iliopsoas. The findings confirmed WNV neuroinvasive disease, despite the prior classification as WNV fever, with limited acute flaccid paralysis (poliomyelitis variant) involving the right lower limb. Laboratory tests to exclude underlying autoimmune disorders were normal, including CBC, CRP, ESR, CMP, ANA, rheumatoid factor (RF), and total creatine kinase (CK). Repeat cytokine panel performed 5 months after onset of symptoms was normal. At that time, many of her symptoms had improved although she was “not back to normal.” At 9 months follow-up, she still had moderate fatigue, decreased stamina, persistent soreness in legs “not related to the amount of physical activity”, and paresthesias in both legs, “like the ends of fibers are just ‘pinging' continuously.” Dysautonomia symptoms also improved, although she had residual focal areas of hyperhidrosis and persistent “trouble adjusting my internal temperature.”

#### Patient 4

A 38-year-old woman developed an acute illness diagnosed as WNV fever followed by persistent symptoms for 36 months, including severe post-exertion fatigue, generalized weakness, imbalance, muscle aches (myalgias), joint pain (arthralgias), low back pain, generalized dysesthesias (“my whole body feels inflamed”) superimposed on subjective low-grade fever, facial flushing, worsening migraines, poor sleep, and dysautonomia symptoms (bladder incontinence, constipation, increased anxiety attributed to a persistent “stressed out feeling” and palpitations). Cognitive issues included difficulty concentrating, memory lapses and increased irritability. She was a former college athlete and a physical therapist in good health prior to WNV with only occasional migraine headaches. The cytokine panel, performed 36 months after onset of symptoms, showed elevated TNF-α, IFN-γ, and IL-13, with equivocal S100B (Table S1). Pertinent normal laboratory tests included CBC, CRP, ESR, ANA, extractable nuclear antigen (ENA), RF, CK, human immunodeficiency virus (HIV), syphilis serology, CMP, Lyme serology, monoclonal protein study, thyroid functions, and paraneoplastic panel. Neurological examination, including mini-mental status assessment, was unremarkable. Following WNV infection, the patient failed treatment by doctors in multiple specialties (internal medicine, cardiology, gastroenterology, rheumatology, and neurology) directed at symptoms arising from organ systems in their respective fields.

In all subjects, we ordered a trial of TNF-α-blocking agents after discovering that WNV post-infectious symptoms were associated with pathological levels of TNF-α. However, immunotherapy was denied by the insurance companies because this treatment did not meet their medical necessity guidelines and peer-reviewed medical literature did not support the use of these agents for treatment of TNF-α symptoms related to a WNV diagnosis. Demographic, laboratory and chief persistent symptoms of the 4 cases are shown in Table S1. Fatigue and pain were the most common persistent symptoms, shared by all 4 subjects. The pain primarily presented as arthralgias (*n* = 4), myalgias (*n* = 3), or generalized or multifocal pain (*n* = 2). Three of 4 also complained of generalized or focal weakness, imbalance, and anxiety. Two had symptoms of persistent low-grade fever, headaches, difficulty concentrating, tremors, sleep disruption, and symptoms of dysautonomia (palpitations, irregular heart rate, postural intolerance, orthostatic tachycardia, diarrhea, hyperhidrosis, and changes in bowel or bladder function).

## Discussion

We present four WNV patients with persistent post-infectious symptoms associated with elevated levels of TNF-α and other proinflammatory cytokines that promote chronic inflammation. The present case series extends previous reports of elevated cytokines in WNV survivors (16, 18, 20) and includes three original observations. First, the clinical data provide evidence that WNV infection in humans induces a significant upregulation of TNF-α. This is not surprising since previous studies in mice and cell cultures have demonstrated essential protective roles for TNF-α and other proinflammatory cytokines against *acute* WNV infection (10, 12–14) Moreover, high TNF-α levels have been reported in other flavivirus human infections, including dengue (21, 22), Zika virus (23, 24), and Japanese encephalitis virus (JEV) (25). Children infected with dengue virus showed significantly higher serum levels of TNF-α, with the highest levels in those with severe dengue disease (21), formerly “dengue shock syndrome” and “dengue hemorrhagic fever.” In fact, the potentially critical causative role of TNF-α and the associated “cytokine storm” in severe dengue disease and other viral diseases has long been recognized (26). In fatal cases of Zika fetal syndrome, a significantly increased expression of TNF-α, IFN-γ, and other proinflammatory cytokines has been found in the meninges, perivascular region, and parenchyma of microcephalic brains (23, 24). Japanese encephalitis virus (JEV) infection also significantly elevates expression of TNF-α and other pro-inflammatory cytokines in animals and cell cultures, leading to neuroinflammation and neuronal death (25). Hence, it is not surprising that we now add TNF-α to the list of proinflammatory molecules that are upregulated in humans following WNV infection. Second, the observations suggest that elevated levels of TNF-α and other antiviral cytokines may persist long after the virus has been cleared by effective innate and adaptive immune responses, even in non-neuroinvasive disease cases classified as WNV fever. Indeed, infectious WNV cannot be isolated from humans with a normal immune system following the production of WNV-specific IgM antibodies (3–5), which are usually detectable 3 to 8 days after onset of clinical illness (1). In our case series, high TNF-α values were detected at weeks 8, 10, 12, and 36 months post-onset of illness. Repeat cytokine panels at months 5, 20, and 21 after onset of symptoms showed normal TNF-α values, suggesting that TNF-α may play a more prominent role in *initiating* rather than *maintaining* the proinflammatory environment. In fact, in one major study, 44 of 140 WNV patients (31%) that reported prolonged (> 6 months) post-infectious symptoms, with an average symptom duration of 5 years, had significantly elevated pro-inflammatory proteins that included IL-2, IL-6, IL-12p70, granulocyte macrophage colony stimulating factor, IFN-γ, and IFN-γ-inducing protein 10, but not TNF-α (16). Moreover, in case 2, soluble IL-2 receptor (CD 25), an established inflammatory marker that reflects ongoing immune activation and inflammation in multiple human autoimmune diseases (19), increased from 1220 to 1571 (normal <1033 pg/mL) over the ensuing 17 months despite normalization of TNF-α. These examples are in agreement with TNF-α being most active in orchestrating the innate immune response that initiates an inflammatory cascade (13, 17). However, in case 4, the initial cytokine panel performed 36 months after onset of illness showed elevated TNF-α, IL-13, IFN-γ, and equivocal S100B values, suggesting that in some cases TNF-α may remain elevated for years post-infection. Third, the clinical evidence supports the contention that TNF-α signaling may contribute to the persistent symptoms that have plagued WNV survivors since WNV gained entry into North America in 1999. Indeed, we ordered the comprehensive cytokine panel to better understand the pathophysiology of the protracted symptoms and TNF-α was the *only* cytokine elevated in all 4 subjects. In case 3, a “flare-up” of colitis may have contributed to the elevated TNF-α and IFN-γ. However, the colitis was stable until WNV infection and the persistent symptoms that brought this patient and others to medical attention were typical of WNV infection rather than coexisting disorders. Importantly, the post-infectious WNV environment has been reported to promote or amplify various diseases that have a presumed autoimmune pathogenesis, including Guillain-Barre syndrome, (27) other demyelinating neuropathies (28), *de novo* myasthenia gravis (29), transformation of stable ocular myasthenia gravis to myasthenic crisis (30), and stiff-person syndrome (31).

TNF-α is widely recognized as a proinflammatory molecule that serves as an important component of neuroinflammation and systemic inflammation in many autoimmune diseases (11, 13, 17). TNF-α and other antiviral proinflammatory cytokines initially protect against WNV infection in animal models (12–14). Treatment of cells with a neutralizing anti-TNF-α monoclonal antibody initially decreases survival in mice during the acute WNV infection (11, 15), when antiviral cytokines are needed to clear the virus. In contrast, administration of the same anti-TNF-α treatment at a later stage of infection results in a significant reduction of WNV-mediated neuronal death, suggesting that persistence of such proinflammatory mediators may play a major role in the pathogenesis of WNV infection in the CNS (11, 15). In a longitudinal cross-sectional study to assess changes in neurological status over time, neurological examinations were performed 1–3 years and 8–11 years post-WNV infection in 60 WNV patients (35 encephalitis, 14 meningitis, 11 WNV fever) (32). By the time of the second assessment, 36% of encephalitis, 33% of meningitis, and 57% of WNV fever cases had developed new neurological complications. The authors concluded that long-term neurological deficits may develop years later, regardless of the severity of the initial WNV infection (32). The clinical data from this longitudinal study, in conjunction with several studies showing that proinflammatory cytokines can be chronically elevated in WNV survivors (16, 18, 20), support the concept of a pathogenic role for persistent virus-specific immunity. Importantly, the receptors of cytokines are expressed constitutively throughout the CNS, including neurons, thereby rendering them sensitive to cytokines even at very low levels (10). IFN-γ, which was elevated in cases 3 and 4, also induces proinflammatory chemokines that are important triggers of inflammation in the brain (10, 33). The early up-regulation of these chemokines in the CNS coincides with the up-regulation of TNF-α at the same site in a rodent model of WNV infection (33). Collectively, the evidence supports the concept that an extended elevation of TNF-α and other proinflammatory cytokines in WNV survivors, as in other autoimmune and inflammatory diseases, plays a causal rather than a coincidental role in the development of protracted or delayed symptoms.

In all four patients a trial of immunotherapy with TNF-α-blocking agents was denied by respective insurance companies due to not meeting medical necessity guidelines and because peer-reviewed medical literature did not support the use of these agents as safe or effective for treatment of TNF-related symptoms with a WNV diagnosis. The denials occurred despite patients' developing a plethora of symptoms that are generally recognized as classic for TNF-α-related pathogenesis in autoimmune diseases such as rheumatoid arthritis and psoriatic arthritis, including fatigue, weakness, arthralgias, and generalized or multifocal pain.

## Conclusion

These cases provide evidence that WNV-induced TNF-α signaling, which modulates the expression of multiple inflammatory mediators, may persist in some WNV survivors and become detrimental, contributing to a *post-infectious pro-inflammatory state* linking chronic neuroinflammation with WNV disease pathogenesis. In such cases, persistent or delayed neurological symptoms following the production of WNV neutralizing antibodies is more likely to reflect secondary injury from the downstream cascade of excitotoxic events and the secondary wave of neuroinflammation rather than neuronal loss per se. Greater awareness is needed that this proinflammatory state may be potentially treatable with drugs that lessen the inflammatory microenvironment. At present, no specific therapy has been approved for human use in WNV infection and patients and family members are often informed that only supportive care is available. However, the merging knowledge about the immune-mediated post-infectious pathogenesis of WNV infection has direct therapeutic implications. Appropriate clinical trials seem warranted to determine if TNF-α inhibitors or other immunosuppressive therapies can control this immunological cascade to improve patient outcomes.

## Data Availability Statement

The datasets generated for this study are available on request to the corresponding author.

## Ethics Statement

The studies involving human participants were reviewed and approved by the Institutional Review Board for human Research at Methodist Rehabilitation Center, Jackson, MS, USA. The patients/participants provided their written informed consent to participate in this study. Written informed consent was obtained from the individual(s) for the publication of any potentially identifiable images or data included in this article.

## Author Contributions

AL, SS, and DS made substantial contributions to the acquisition and analysis of the clinical data for the work. MG and BG made substantial contributions to the conception of the work. PV and FB made substantial contributions revising the work and adding critically important biochemical intellectual content. Provide approval for publication of the content and agreed to be accountable for all aspects of the work in ensuring that questions related to the accuracy or integrity of any part of the work are appropriately investigated and resolved. All authors provide approval for publication.

## Conflict of Interest

The authors declare that the research was conducted in the absence of any commercial or financial relationships that could be construed as a potential conflict of interest.
