# Supplementary material for: Tumor Necrosis Factor-Alpha Signaling May Contribute to Chronic West Nile Virus Post-infectious Proinflammatory State
Source: Front Med (Lausanne). 2020 Apr 30;7:164. doi: 10.3389/fmed.2020.00164 (PMC7203783; doi:10.3389/fmed.2020.00164)
Supplement: Supplementary file 1 [file Table_1.pdf]

Table 1: Demographic, laboratory and chief persistent symptoms

Age/Sex	Interval*	Cytokines	Persistent symptoms
49, M	8 wks	TNF-α 56 pg/mL<br>S100B 81 ng/L	Fatigue, arthralgias, generalized and focal weakness, anxiety, imbalance, generalized and multifocal pain, tremors, restlessness.
53, F	10 wks	TNF-α 28 pg/mL<br>IL-2R CD25-sol 1220 pg/mL<br>S100B 81 ng/L	Fatigue, arthralgias, myalgias, anxiety, imbalance, low grade fever, headaches, difficulty concentrating, insomnia, dizziness, limb paresthesias, depression.
34, F	12 wks	TNF-α 39 pg/mL<br>INF-ɣ 17 pg/mL	Fatigue, arthralgias, myalgias, right leg weakness with involuntary "jerks", hand tremors, legs shaking, total body hyperexcitability, impaired hand/eye coordination, dysautonomia: palpitations, irregular heart rate, postural intolerance, orthostatic tachycardia, diarrhea, hyperhidrosis and "trouble adjusting my internal temperature."
38, F	36 mo.	TNF-α 54 pg/mL<br>INF-ɣ 11 pg/mL<br>IL-13 8 pg/mL<br>S100B 90 ng/mL	Fatigue, arthralgias, myalgias, generalized weakness, anxiety, increased irritability, imbalance, low back pain, generalized dysesthesias ("my whole body feels inflamed"), low-grade fever, headaches, difficulty concentrating, memory lapses, facial flushing, poor sleep, and dysautonomia: palpitations, bladder incontinence, constipation.

* Interval between onset of West Nile virus symptoms and cytokine panel collection.

Abbreviations: TNF-α, Tumor necrosis factor alpha; S100B, S100B protein; IL-2R CD25-sol, interleukin-2 receptor CD 25 soluble protein; INF-ɣ, Interferon gamma; IL-13, Interleukin-13.

Reference Range:

TNF-α ≤ 22 pg/mL.

S100B ≤ 96 ng/L; Equivocal 80 - 95 ng/L

IL-2R CD25-sol ≤ 1033 pg/mL

INF-ɣ ≤5 pg/mL
